# Thermal Gradient Effects on Redox Evolution and Volatility-Driven Fractionation in Ternary U/Ce/Cs Condensates

**DOI:** 10.1021/acs.analchem.5c07929

**Published:** 2026-04-24

**Authors:** Rakia Dhaoui, Emily N. Weerakkody, Timothy P. Rose, Batikan Koroglu, Enrica Balboni

**Affiliations:** Physical and Life Sciences Division, 536768 Lawrence Livermore National Laboratory, Livermore, California 94550, United States

## Abstract

Understanding how thermal history influences redox evolution and chemical fractionation is essential for characterizing high-temperature condensation in complex materials, including nuclear debris. Here, we tested the hypothesis that distinct thermal regimes in a plasma flow reactor influence redox pathways and elemental partitioning in ternary U/Ce/Cs systems. A configurable plasma flow reactor was modified with an external tube furnace to impose two distinct thermal gradients: continuous ambient cooling and a furnace-assisted thermal hold-up near 1400 K followed by rapid cooling. Transmission electron microscopy characterized phase identity, morphology, and nanoscale element distributions, while inductively coupled plasma-mass spectrometry quantified bulk elemental ratios. Across both thermal regimes, uranium and cerium condensed as UO_2_ and CeO_2_ as dominant refractory oxide products. Uranium partially oxidized to α-UO_3_ during extended ambient cooling, while furnace-assisted hold-up preserved UO_2_ and produced partial reduction of cerium to Ce_2_O_3_. Cesium remained volatile upstream and condensed later in the reactor, forming Cs_2_O and Cs-uranate phases with the highest incorporation after thermal hold-up. Bulk ICP-MS measurements supported these observations. U/Ce ratios remained comparatively stable and Cs displayed delayed and apparent transient enrichment that matched the nanoscale measurements. This integrated approach provides a quantitative method for linking thermal gradients to redox evolution and volatility-driven fractionation. These results show how the plasma flow reactor can identify where equilibrium descriptions remain adequate and where kinetic effects from residence time and temperature history must be considered when interpreting condensation behavior in multicomponent systems.

## Graphical abstract

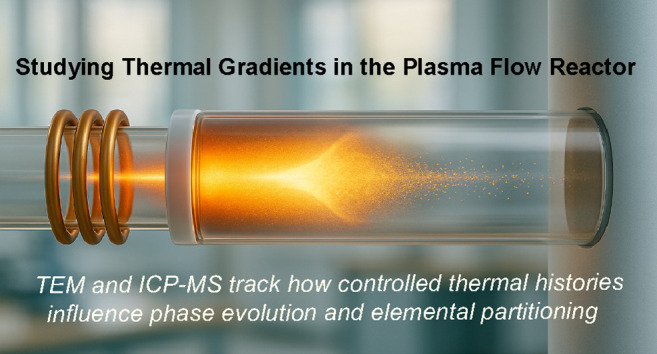

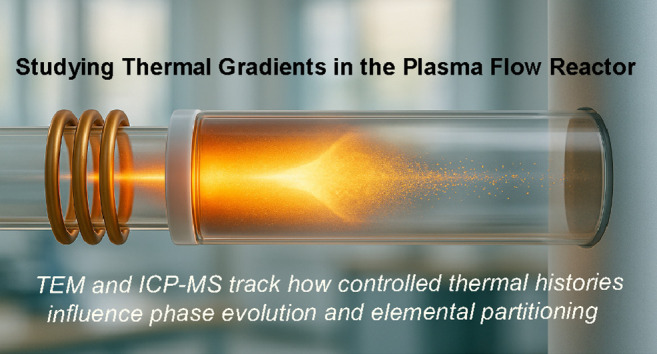

## Introduction

Condensation in high-temperature environments controls the composition and structure of many complex particles, including nuclear debris. During a nuclear detonation or reactor accident, materials vaporize at several thousand kelvins and then cool rapidly as they mix with surrounding gases and solids.^1,2^ Refractory and volatile elements condense at different temperatures and residence times, and these differences govern radionuclide transport, deposition, and long-term environmental behavior.^3,4^ Fallout forms under steep thermal gradients, which produce strong chemical heterogeneity across individual particles. This heterogeneity complicates hazard prediction and nuclear forensic analysis. Classical fallout models, which were largely developed from midtwentieth-century atmospheric tests, assume equilibrium-controlled oxide formation.^5–8^ These models capture the dominant refractory oxides, but field samples show significant deviations in the distributions of volatile and refractory species.^8,9^ Differences in radionuclide ratios and phase assemblages indicate that condensation depends not only on equilibrium chemistry but also on temperature history,^10,11^ oxygen availability,^8,9^ and matrix composition.^4,9,12,13^ Addressing these limitations requires an experimental platform that reproduces high-temperature vaporization while isolating thermal history and chemical environment in a controlled manner.

Here, we customized a plasma flow reactor to impose controlled thermal gradients and evaluate how they influence redox evolution and vapor–condensate partitioning in a ternary U/Ce/Cs matrix. The reactor maintains plasmas above 5000 K and allows programmable control of cooling profiles and oxygen fugacity.^14–17^ In this work, we examine two contrasting thermal regimes: continuous ambient cooling and a furnace-assisted thermal hold-up that imposes an extended high-temperature interval near 1400 K before quenching. We describe the furnace-assisted condition as near-equilibrium because the increased residence time and thermal uniformity promote vapor–solid reactions that move closer to thermodynamic steady state. Previous studies in this reactor showed that uranium oxide speciation is sensitive to both oxygen fugacity and cooling history, stabilizing as either UO_2_ or α-UO_3_ depending on the temperature and redox conditions.^15,18–21^ Similar work on cerium showed that while CeO formation scales with oxygen fugacity at high temperature, CeO_2_ is the primary condensed phase and exhibits vapor-phase aggregation behavior distinct from uranium.^22^

The present study builds on this foundation by examining redox evolution, volatility, and condensate formation in ternary U/Ce/Cs condensates under controlled thermal gradients. The U/Ce/Cs matrix was selected for its chemical relevance and experimental tractability. Uranium provides a refractory actinide with multiple oxidation states.^9^ Cerium serves as a plutonium surrogate with parallel redox behavior.^22–24^ Cesium is a volatile fission product that condenses late and responds strongly to temperature history.^3,25–27^ We tested the hypothesis that distinct thermal gradients alter redox evolution and volatile cesium partitioning during ternary condensation. To evaluate this hypothesis, we combined nanoscale analysis by transmission electron microscopy (TEM) with bulk fractionation profiles from inductively coupled plasma-mass spectrometry (ICP-MS). The TEM methods, including high-resolution TEM (HRTEM), selected area electron diffraction (SAED), high-angle annular dark-field scanning TEM (HAADF-STEM), and energy-dispersive X-ray spectroscopy (STEM-EDS), resolved crystalline phases, particle morphologies, and nanoscale element distributions, while ICP-MS quantified bulk partitioning and linked fractionation trends to nanoscale structures. This dual-scale approach provides a quantitative method for determining how thermal gradients impact redox evolution, volatility-driven fractionation, and phase development in a controlled multicomponent matrix. It also strengthens the capability of the reactor to characterize high-temperature condensation processes in complex chemical systems.

## Experimental Section

### Plasma Flow Reactor Design and Operation

A plasma flow reactor was used to generate high-temperature vapors and controlled cooling profiles for ternary U/Ce/Cs mixtures. The reactor consists of a 2 cm outer-diameter inductively coupled plasma torch connected to a 4 cm fused-quartz reaction tube, which was operated as a steady-state, continuous-flow platform for aerosol processing.^14,15,17^ Precursor solutions containing uranyl, cerium, and cesium nitrates in a 1:1:1 atomic ratio were delivered through the central torch channel with a concentric nebulizer and spray chamber. Argon carrier gas and annular stabilization flows maintained plasma stability and limited heating of the quartz walls. As shown in Figure 1, a resistively heated tube furnace was mounted around the quartz tube to introduce a controlled high-temperature interval. With the furnace off, vapors cooled continuously by convection and radiation. With the furnace on, vapors entered a thermal plateau near 1400 K before final quenching. In both regimes, aerosols were vaporized in the plasma core at approximately 5000 K before cooling and condensation downstream. For bulk ICP-MS quantification, condensates were collected at nine axial positions between 20 and 90 cm. Experiments were run in triplicate for both ambient and furnace-assisted high-temperature conditions, resulting in a total of 54 runs to verify reproducibility. For nanoscale analysis by TEM, separate collections were performed at three representative positions (25, 70, and 90 cm) when the furnace was present, and two positions (25 and 90 cm) when the furnace was absent (i.e., ambient cooling).

**Figure 1:**
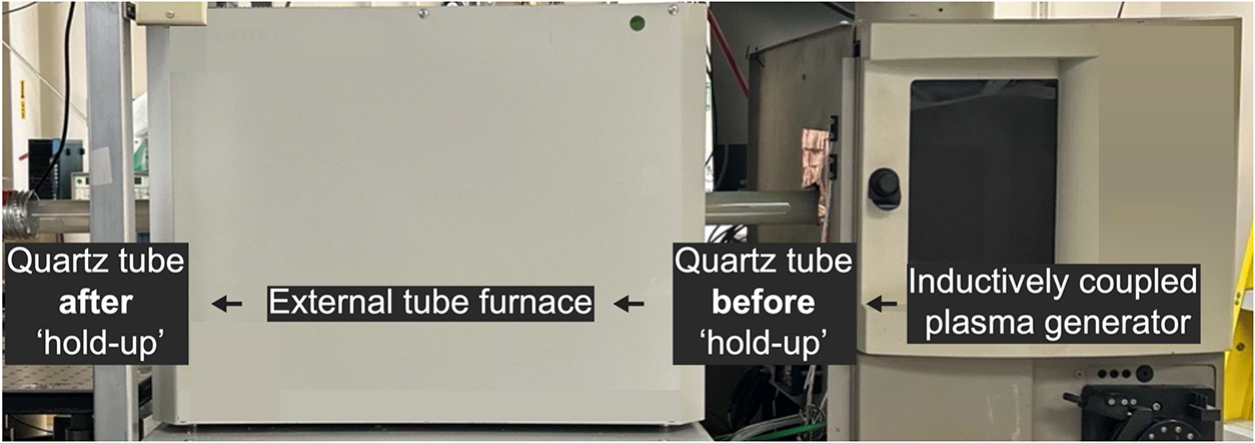
Annotated photograph of the modified plasma flow reactor showing the inductively coupled plasma generator, quartz reaction tube, and resistively heated furnace. Black arrows indicate the direction of plasma flow through the reactor (*right to left*).

### Thermal Profiling and Regimes

Axial temperature profiles defined two thermal regimes: continuous ambient cooling and furnace-assisted hold-up. Under ambient conditions, vapors cooled gradually downstream by convection and radiation. With furnace assistance, a thermal plateau near 1400 K was introduced between approximately 40 and 75 cm before rapid quenching. Axial gas temperatures were measured to define the thermal environment experienced by the condensates. Calibration procedures and diagnostic methods have been described in previous work.^14,17,19^ Briefly, the plasma core reached ∼5000 K and cooled along the axis, yielding an average thermal gradient of 75 K ms^–1^. Axial temperatures were measured using optical emission spectroscopy of thermally excited argon and cerium lines. Line intensities were evaluated by the Boltzmann method to obtain spatially resolved temperature profiles.^19,22^ These complementary diagnostics produced continuous temperature maps along the reactor axis, allowing the thermal gradients associated with ambient cooling and furnace-assisted hold-up to be quantified.

### Condensate Sampling and TEM Grid Preparation

Condensates for ex situ nanoscale analysis were collected using alumina sampling probes fitted with PELCO holey SiN*_x_* TEM support films (200 nm membrane, 3 mm frame). Each probe was inserted to the desired axial position and exposed to the vapor stream for 3 min. Aqueous U/Ce/Cs nitrate solutions (1:1:1 atomic ratio) were injected into the plasma during sampling to maintain constant vapor composition. Condensates were collected at three axial positions: 25 cm (prefurnace cooling), 70 cm (within the furnace), and 90 cm (postfurnace cooling). Each sampling interval lasted 3 min and was conducted in triplicate. The resulting particle density was sufficient for TEM analysis. Particle deposition occurred primarily below 1000 K, and the 25 and 90 cm positions produced the highest particle loading suitable for microscopy, consistent with rapid nucleation during the initial and final cooling stages.^19^ Condensates collected at 70 cm correspond to material formed during furnace-assisted thermal hold-up, providing a means to interpret condensation behavior under prolonged high-temperature residence. Samples at this position were analyzed exclusively under furnace-assisted conditions corresponding to the thermal hold-up region. Sampling was not performed at this position under ambient cooling, since condensates collected at 90 cm already provided a direct comparison of products formed before and after the furnace-induced hold-up.

### TEM Imaging and Structural Identification

The TEM analyses were performed using an FEI Titan 80–300 S/TEM operating at 300 kV and equipped with a Super-X G2 energy-dispersive X-ray spectroscopy detector. Bright-field TEM and high-resolution TEM were used to determine particle morphology and lattice structure. Selected-area electron diffraction was used to identify crystalline phases by comparing diffraction patterns with reference data for UO_2_,^28^ α-UO_3_,^29^ U_3_O_8_,^28,30^ CeO_2_,^31^ Ce_2_O_3_,^32^ Cs_2_O,^33^ Cs_2_UO_4_ and Cs_2_U_2_O_7_.^34^ Elemental distributions of U, Ce, Cs, and O were mapped using high-angle annular dark-field scanning TEM coupled with EDS.

### Sample Collection and Preparation for ICP-MS Analysis

For bulk chemical analysis, condensates were collected on acid-washed alumina stubs placed at each axial sampling location. Each stub was digested in 9 M HCl at 80 °C, followed by repeated rinsing and evaporation to recover uranium, cerium, and cesium. Residual matrix and organic components were removed by sequential treatments with concentrated HNO_3_. Final digestates were reconstituted in 2% HNO_3_, reheated to ensure complete solubilization, and transferred to acid-cleaned centrifuge tubes. Digestion fidelity was validated using matrix-matched aqueous standards with identical U:Ce:Cs input ratios. A detailed digestion protocol is provided in the Supporting Information.

### ICP-MS Instrumentation and Quantification

Elemental concentrations were measured using a Thermo Scientific iCAP-Q quadrupole ICP-MS. Calibration curves were prepared from serial dilutions of certified single-element standards (10 μg mL^–1^, High Purity Standards, Charleston, SC). Analytical blanks and calibration checks were included with each measurement sequence. Concentrations are reported per mass of digested sample. Atomic ratios (U/Ce, Cs/Ce, Cs/U) were calculated to construct axial fractionation profiles and assess how thermal gradients affected elemental partitioning during condensation.

## Results and Discussion

### Thermal Regimes for Testing the Role of High-Temperature Residence

The plasma flow reactor (Figure 2a) was modified with a resistively heated tube furnace to impose controlled thermal gradients and to evaluate how temperature history influences condensation in the ternary U/Ce/Cs system. This design enabled two reproducible cooling environments relevant to our central hypothesis. Under ambient operation, vapors cooled monotonically along the reactor axis by convection and radiation (Figure 2b, *blue circles*). With the furnace active, the vapors encountered a thermal plateau near 1400 K between approximately 40 and 75 cm, followed by rapid downstream quenching (Figure 2b, red squares). We hypothesized that this sustained high-temperature residence and subsequent sharp cooling would create a strong thermal gradient that shifts redox evolution and alters the point at which volatile elements begin to condense. These two thermal regimes provide a controlled framework for testing how high-temperature residence affects redox pathways and volatility-driven fractionation during multicomponent condensation. The furnace-assisted regime isolates the influence of extended high-temperature residence, while the ambient regime captures rapid cooling that suppresses equilibration. This contrast defines the basis for interpreting the nanoscale and bulk measurements that follow.

**Figure 2:**
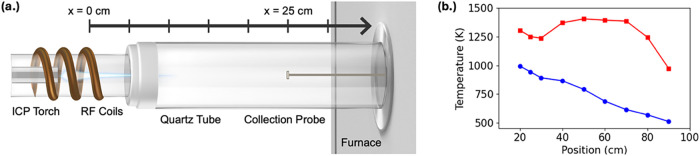
(a) Schematic of the plasma flow reactor setup showing the fused quartz reaction tube, ring-flow injector location (defined as *x* = 0 cm), representative axial sampling positions (e.g., *x* = 25 cm), and external furnace. Flow is depicted left-to-right for clarity. (b) Axial gas temperature profiles under ambient cooling (*blue circles*) and furnace-assisted thermal hold-up (*red squares*).

### Nanoscale Evidence for Thermal-Gradient Control of Redox Evolution and Cesium Incorporation

To evaluate how distinct thermal regimes influence redox evolution and elemental partitioning, we examined condensates collected before, within, and after the furnace-induced thermal plateau. These sampling positions represent three stages of particle development as vapors cool through the reactor. Condensates at 25 cm capture early nucleation during a steep temperature decline. Condensates at 70 cm reflect growth under the sustained high-temperature plateau created by the furnace. Condensates at 90 cm represent late-stage products that have cooled either continuously under ambient conditions or after extended high-temperature residence. This enables controlled comparison of how thermal history shapes particle morphology, phase identity, and nanoscale element distribution.

### Early-Stage Condensation at 25 cm

Condensates collected at 25 cm recorded the earliest stage of particle formation, where temperatures fall rapidly and residence times are short. Across both thermal regimes, bright-field TEM showed small, discrete crystallites with minimal aggregation, which is consistent with rapid nucleation (Figure 3). SAED patterns identified UO_2_ and CeO_2_ as the dominant phases, showing that these refractory oxides form the structural backbone of the condensates. Cesium-bearing phases were not detected at this stage, which is expected given its high volatility.

**Figure 3:**
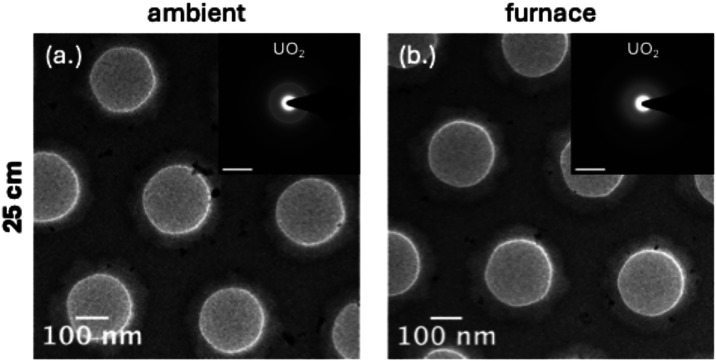
Bright-field TEM images of U/Ce/Cs condensation products collected at 25 cm under (a) ambient and (b) furnace-assisted cooling. Insets show corresponding SAED patterns, with the dominant U-bearing phase highlighted to guide comparison across thermal conditions. Scale bars = 5 nm^–1^.

Because this position marks the beginning of condensation, samples were further examined using HRTEM, HAADF-STEM, and STEM-EDS to resolve features beyond those visible in bright-field imaging and SAED. Under ambient cooling, HRTEM identified lattice spacings consistent with α-UO_3_ and UO_2_ (Figure 4a-i,a-ii, respectively), indicating that uranium underwent partial oxidation during early quenching. HAADF-STEM and EDS mapping (Figure 4b) showed limited mixing between uranium and cerium, with uranium preferentially condensing on cerium-rich domains. Under furnace-assisted conditions, particles retained their discrete morphology but were larger and more spherical (Figure 4c). HRTEM confirmed that uranium condensed exclusively as UO_2_ (Figure 4c-i,c-ii), with no evidence of higher-valence oxides. HAADF-STEM and STEM-EDS maps (Figure 4d) again showed limited U–Ce mixing, with uranium appearing to deposit onto cerium-rich domains. These observations confirm that UO_2_ and CeO_2_ persist as stable near-equilibrium refractory oxides across both regimes.^35,36^ Ambient cooling promotes early oxidation of uranium toward U­(VI), whereas furnace-assisted conditions (∼200 K hotter) stabilize UO_2_ by suppressing oxidation.^19^ Our previous work under analogous ambient conditions with uranium alone produced dominant formation of α-UO_3_.^21^ The difference observed here likely arises from the oxygen-buffering role of cerium, which accommodates oxygen in the Ce­(IV) state,^37,38^ and reduces U–O collisions through redox competition.^18–21^ Cesium was not detected under either regime, consistent with its volatility and delayed condensation downstream.

**Figure 4:**
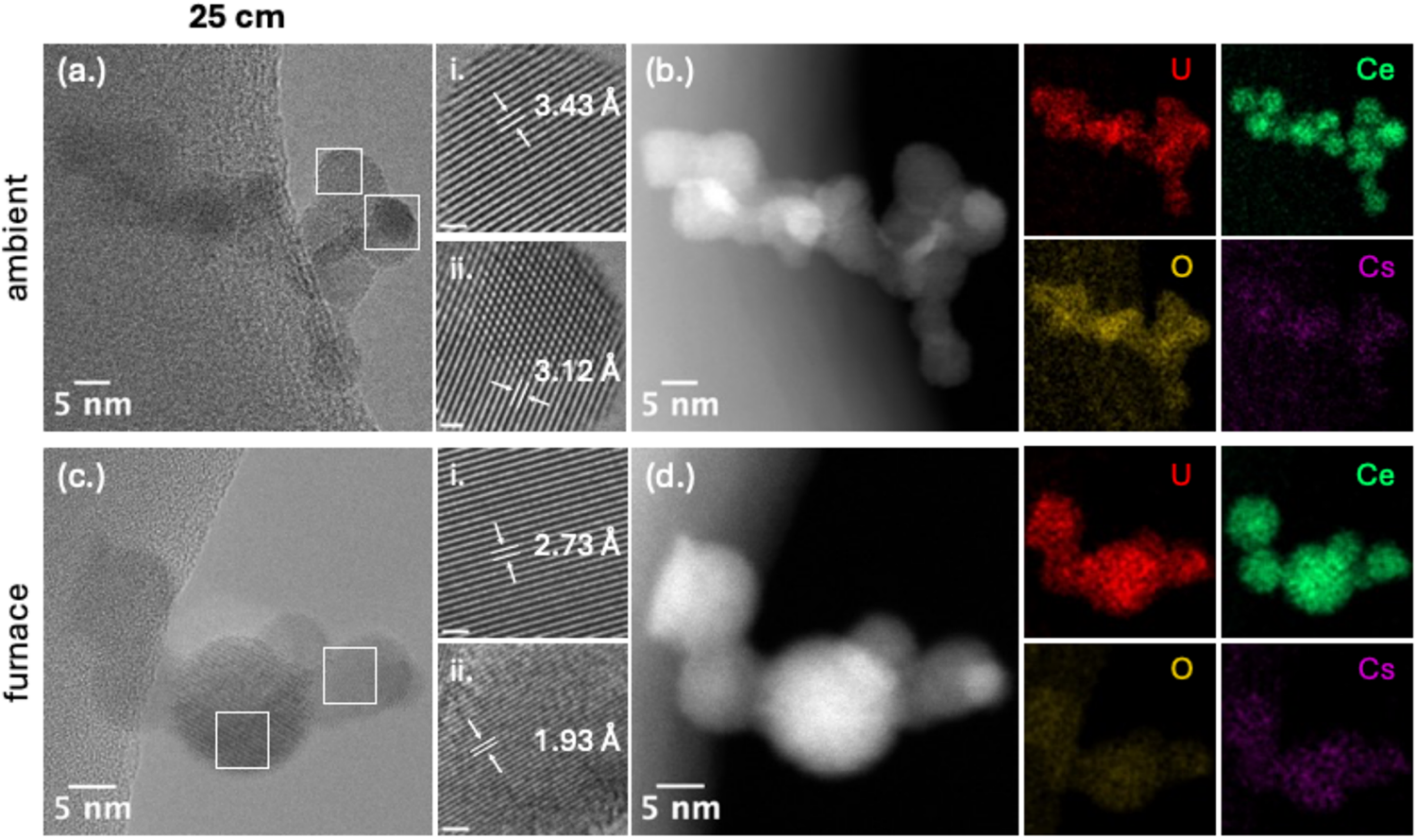
HRTEM, HAADF-STEM, and EDS elemental mapping of U/Ce/Cs condensation products collected at 25 cm under ambient and furnace-assisted cooling. (a) TEM image of particles formed under ambient cooling, with boxed regions shown at higher magnification in (a-i) and (a-ii). (b) HAADF-STEM image and EDS maps of U, Ce, Cs, and O. (c) TEM image under furnace-assisted cooling, with boxed regions shown in (c-i) and (c-ii). (d) Corresponding EDS maps. Scale bars for higher magnification images = 1 nm.

### Intermediate Condensation at 70 cm under Furnace-Assisted Thermal Hold-Up

Condensates collected at 70 cm formed within the furnace-induced thermal plateau and provide a view of particle development under extended high-temperature residence. Sampling at this position was carried out only for the furnace-assisted regime because condensates collected at 90 cm under ambient cooling already serve as the downstream comparison for the ambient or nonfurnace pathway. Bright-field TEM showed sparse particle coverage and discrete faceted crystallites, which is consistent with limited nucleation and slower supersaturation at elevated temperature.^19^ HRTEM images showed lattice fringes consistent with Ce_2_O_3_ (Figure 5a), while the corresponding SAED pattern confirmed Ce_2_O_3_ and CeO_2_ formation. The coexistence of Ce­(III) and Ce­(IV) oxide domains suggests that local redox conditions within the plateau permit reduction but do not fully suppress oxidation, which is consistent with earlier studies of cerium oxide behavior at elevated temperatures.^38–40^ HAADF-STEM and EDS mapping (Figure 5b) showed that cesium incorporation was higher than upstream and localized in the particle core, while uranium and cerium remained spatially distributed across the crystallite. The extent of Cs incorporation is still limited, yet its presence at this stage shows that longer residence at high temperature can promote early Cs interaction with the refractory matrix. These observations support the hypothesis that elevated temperature and longer residence time shift both redox evolution and the onset of volatile-element condensation relative to the ambient cooling pathway.

**Figure 5:**
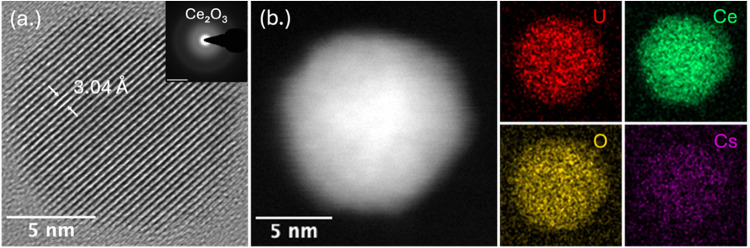
HRTEM, HAADF-STEM, and EDS elemental mapping of U/Ce/Cs condensation products collected at 70 cm under furnace-assisted cooling. (a) TEM image and SAED pattern, with scale bar = 5 nm^–1^. (b) HAADF-STEM image with EDS maps of U, Ce, Cs, and O.

### Late-Stage Condensation at 90 cm

Condensates collected at 90 cm represent late-stage particle formation and show the strongest influence of thermal history. Under ambient cooling (Figure 6a), bright-field TEM showed irregular, aggregated particles that formed through coalescence during continuous cooling. SAED identified UO_2_, α-UO_3_, and CeO_2_, consistent with progressive oxidation of uranium during extended cooling. The presence of α-UO_3_ indicates progressive oxidation of uranium along the continuous ambient cooling pathway. In contrast, under furnace-assisted cooling (Figure 6b), particles remained smaller and discrete, often spherical, with smooth boundaries. SAED identified UO_2_ and CeO_2_ but did not identify α-UO_3_, which indicates that prolonged exposure to the high-temperature plateau suppressed uranium oxidation.

**Figure 6:**
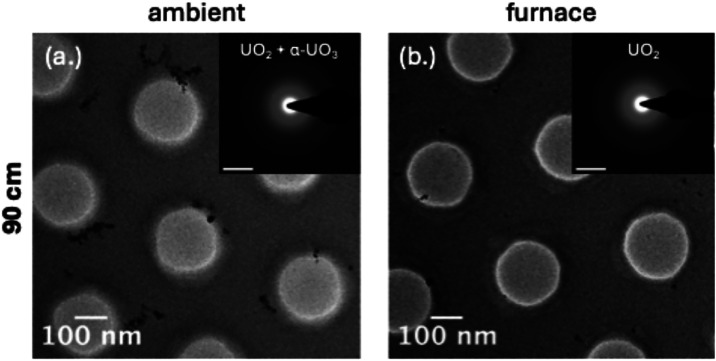
Bright-field TEM images of U/Ce/Cs condensation products collected at 90 cm under (a) ambient and (b) furnace-assisted cooling. Insets show corresponding SAED patterns, highlighting changes in U-bearing oxide phases with thermal history. Scale bars = 5 nm^–1^.

Higher-resolution imaging revealed further divergence in phase chemistry. Under ambient cooling, HRTEM identified domains of Cs_2_O^33^ and Cs_2_UO_4_ ^34^ (Figure 7a-i,a-ii, respectively). The appearance of Cs_2_O requires careful interpretation. Direct CsO_2_ condensation at these temperatures cannot be excluded at ∼800–900 K (the approximate temperature at 90 cm), where CsO_2_ vapor pressures remain appreciable. However, the concurrent presence of Cs_2_UO_4_ and its reported thermal instability at similar temperatures^41,42^ also supports Cs_2_O formation as a byproduct through partial degradation of Cs_2_UO_4_ during downstream cooling. STEM-EDS maps (Figure 7b) showed more extensive U–Ce mixing than at 25 cm, although uranium still condensed largely independently. Cesium enrichment was modest and localized near uranium-rich regions, suggesting it remained in the vapor phase through most of the reactor and condensed in limited amounts. Under furnace-assisted conditions, HRTEM further identified Cs_2_U_2_O_7_ and minor Ce_2_O_3_ formation (Figure 7c-i,c-ii). STEM-EDS maps (Figure 7d) showed more uniform incorporation of cesium across the condensed phase. These observations indicate that extended high-temperature residence promoted partial reduction of cerium and stabilized the more Cs-rich uranate, Cs_2_U_2_O_7_.

**Figure 7:**
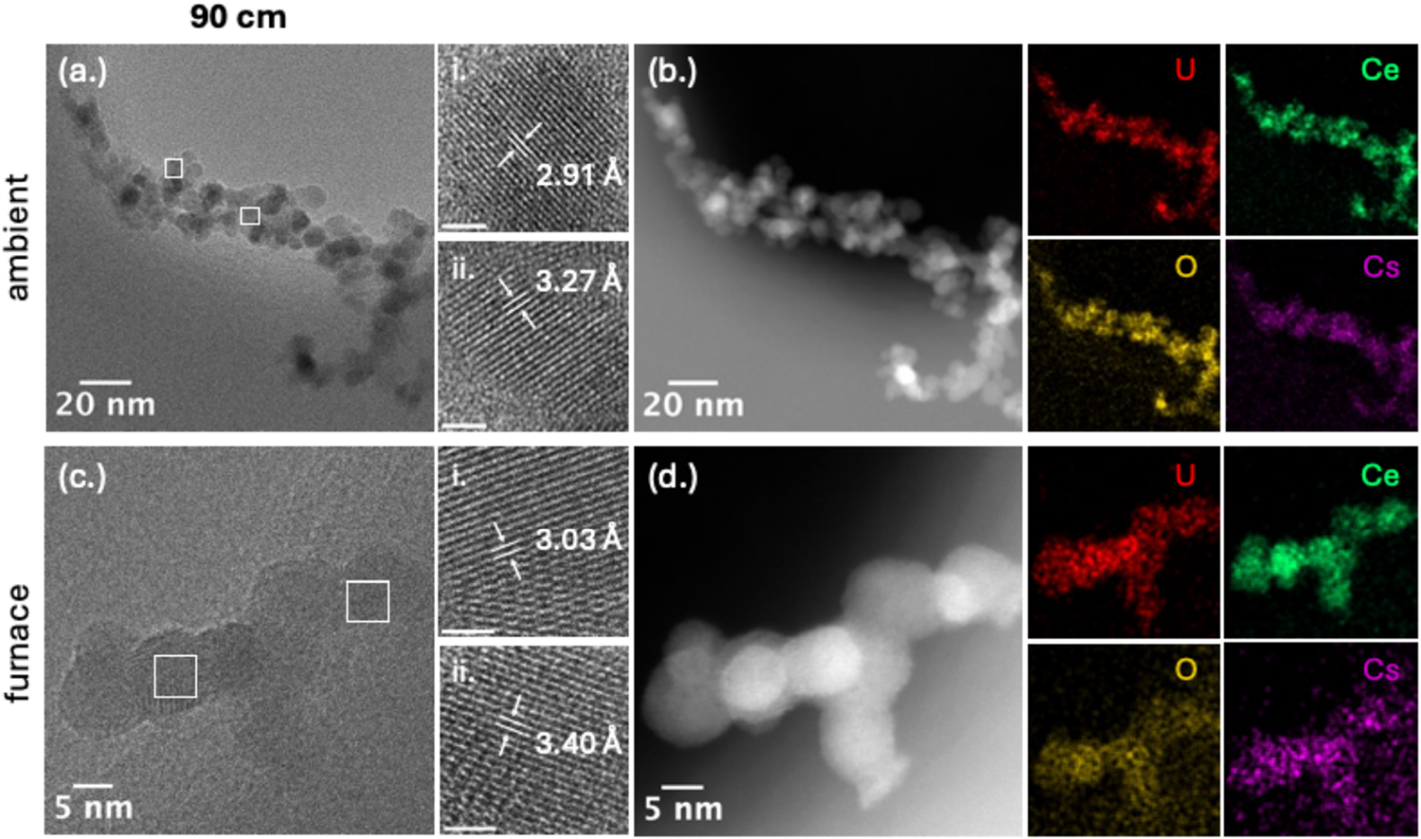
HRTEM, HAADF-STEM, and EDS elemental mapping of U/Ce/Cs condensation products collected at 90 cm under ambient and furnace-assisted cooling. (a) TEM image of aggregates formed under ambient cooling conditions, with boxed regions shown at higher magnification in (a-i) and (a-ii). (b) HAADF-STEM image with EDS maps of U, Ce, Cs, and O. (c) TEM image of condensates formed under furnace-assisted conditions, with boxed regions shown in (c-i) and (c-ii). (d) Corresponding EDS maps. Scale bars for higher magnification images = 1 nm.

The structural diversity observed under furnace-assisted conditions was also greater than under ambient cooling. TEM imaging showed spherical, core–shell, and droplet-like particles containing polycrystalline domains interspersed with amorphous regions (Figure 8). HRTEM confirmed Cs_2_UO_4_ and Cs_2_U_2_O_7_ formation (Figure 8a-i,a-ii),^34,34^ further supporting higher formation of mixed Cs–U oxides after thermal hold-up. Bright-field images also revealed stratified architectures of Cs_2_UO_4_, UO_2_, and amorphous regions (Figure 8b,c), indicating that extended high-temperature residence also promotes structural reorganization.

**Figure 8:**
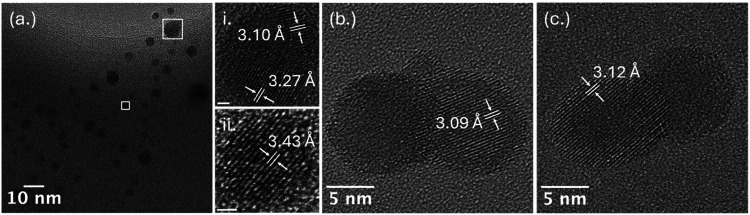
TEM images of (a) droplet and (b, c) spherical condensates formed under furnace-assisted conditions. White boxes indicating regions imaged at higher magnification (a-i, ii), scale bars = 1 nm.

### Bulk ICP-MS Analysis: Elemental Fractionation across Thermal Gradients

Bulk ICP-MS measurements were used to quantify how thermal history influenced U, Ce, and Cs partitioning during condensation. Condensates collected along the reactor were analyzed to determine Cs/Ce, U/Ce, Cs/U, and Ce/U ratios. Because the injected aerosol composition was held at a 1:1:1 atomic ratio, deviations from unity provide a direct indicator of fractionation driven by volatility, residence time, and local temperature. Under ambient cooling (Figure 9a), the U/Ce ratio remained stable across the reactor axis, fluctuating narrowly between 0.9 and 1.1. This near-stoichiometric behavior indicates co-condensation, consistent with the similar volatilities of U and Ce. In contrast, Cs/Ce ratios ranged from about 0.6 to 1.9 and increased steadily with decreasing temperature. This trend reflects the higher volatility of cesium and its delayed incorporation into the condensed phase relative to uranium and cerium. Furnace-assisted cooling produced a stronger fractionation signature (Figure 9b). Cs/Ce ratios reached values as high as 25 immediately downstream of the furnace plateau. This apparent enrichment indicates that extended residence at high temperature limited early incorporation of cesium and promoted stabilization of Cs during quenching. Despite these large variations in cesium behavior, U/Ce ratios remained comparatively stable under furnace-assisted conditions, varying within a factor of ∼2 and indicating broadly coupled condensation of uranium and cerium.

**Figure 9:**
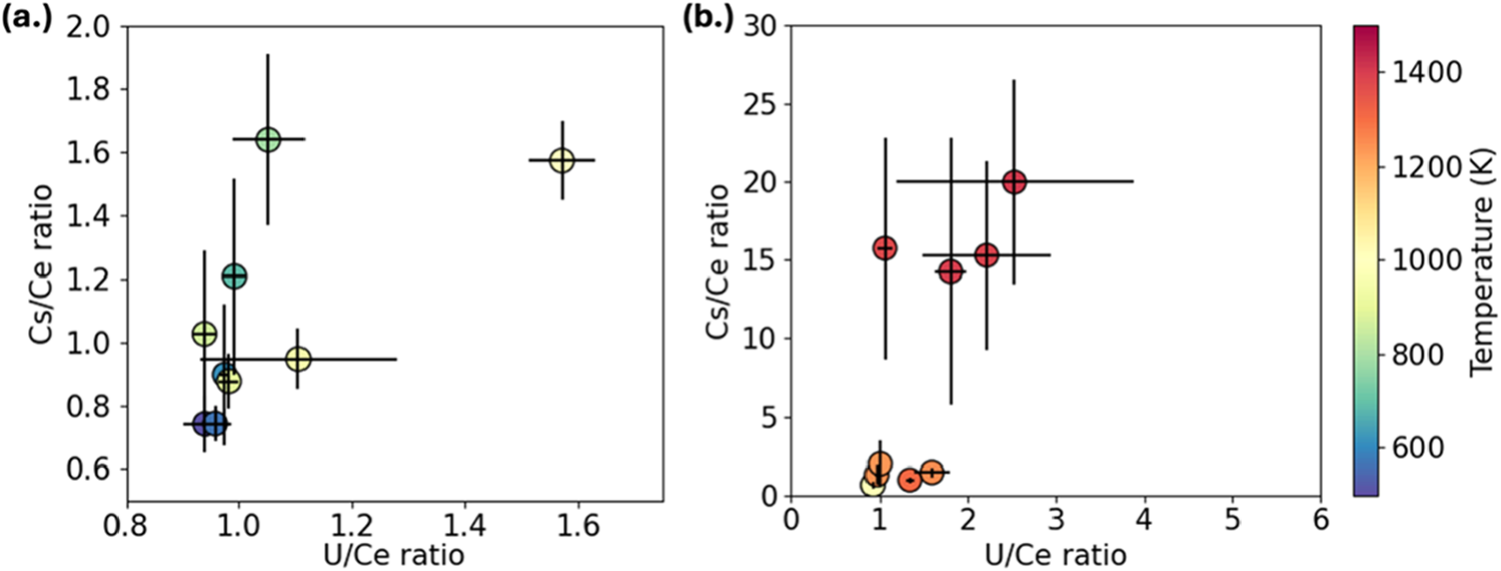
ICP-MS results showing Cs/Ce and U/Ce ratios as a function of temperature. Colormaps of U/Ce vs Cs/Ce atomic ratios for the (a) ambient cooling condition and (b) furnace-assisted condition. Error bars represent one standard deviation from three independent replicate experiments (*n* = 3).

To better visualize the evolution of fractionation along the reactor axis, we examined axial Cs/Ce and U/Ce trends with temperature superimposed (Figure 10). Under ambient cooling (Figure 10a), Cs/Ce ratios increased with decreasing temperature before stabilizing below ∼800 K. U/Ce ratios remained close to 1.0 throughout, indicating that uranium and cerium condensed together in near-stoichiometric proportions. In contrast, furnace-assisted cooling (Figure 10b) showed pronounced deviations. Within the thermal plateau, Cs/Ce ratios rose sharply, peaking around 50 cm, before converging back to unity at 90 cm. This spatially confined enrichment aligns closely with TEM observations of delayed Cs condensation and subsequent incorporation into Cs-uranate phases. Across both regimes, U/Ce ratios remained constant along the axis. This stability confirms that uranium and cerium respond similarly to the thermal gradient, while cesium responds more strongly to high-temperature residence.

**Figure 10:**
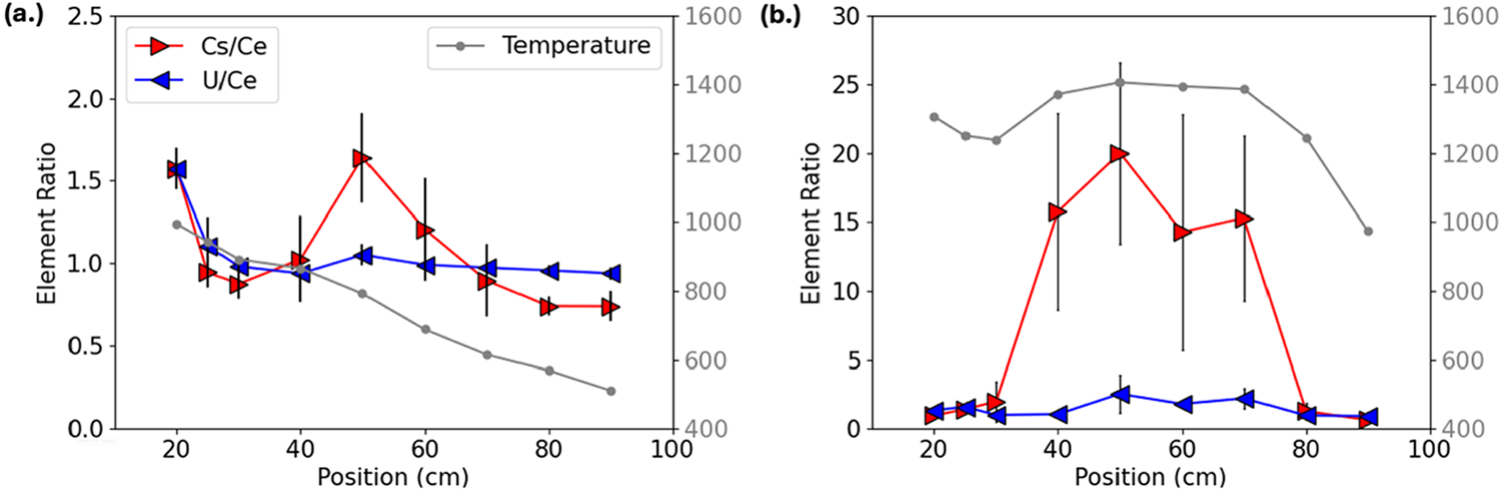
Axial trends in Cs/Ce (*red forward arrows*) and U/Ce (*blue reverse arrows*) elemental ratios with temperature overlay (*gray points*) for (a) ambient and (b) furnace-assisted conditions. Error bars represent one standard deviation from three independent replicate experiments (*n* = 3).

Notably, in both Figures 9 and 10, much higher apparent ratios (e.g., Cs/U > 15) were observed. These elevated values likely reflect the preferential deposition of refractory uranium and cerium oxides onto the cooler reactor walls upstream of the collection points, which lowers the measured U and Ce concentrations relative to Cs. When normalized to these diminished concentrations, cesium appears artificially enriched, inflating Cs/U and Cs/Ce ratios. Such artifacts highlight the need to interpret extreme values within the context of vapor–wall interactions and deposition losses. Even accounting for this effect, the transient enrichments remain consistent with kinetic stabilization of Cs-bearing phases identified by TEM.

### Cross-Scale Interpretation of Redox Evolution and Fractionation

The modified PFR setup described in this work provides a controlled way to evaluate how thermal gradients shape condensation pathways in a multicomponent system. Ambient cooling samples a regime where reactions compete with rapid quenching. Furnace-assisted cooling introduces a sustained high-temperature thermal regime that allows vapor–solid reactions to move closer to steady state before final condensation. We hypothesized that these contrasting regimes would shift the redox evolution of uranium and cerium and would delay or advance cesium incorporation. The combined use of TEM and bulk ICP-MS provides a dual-scale framework to test this hypothesis and to identify where near-equilibrium descriptions remain adequate and where kinetic effects dominate condensate evolution in this simplified system.

Across the reactor, the TEM results show that uranium and cerium condensed early as UO_2_ and CeO_2_. These refractory oxides form the structural backbone of the system under both thermal regimes. Given the similar ionic radii of U­(IV) and Ce­(IV) in 8-fold coordination, partial substitution within fluorite-type domains is plausible and may contribute to the observed nanoscale colocation of U and Ce. However, confirming cation substitution would require higher-resolution structural or spectroscopic analysis. At 25 cm, particles remained small and discrete. Under ambient cooling, uranium displayed partial oxidation to α-UO_3_, which reflects rapid oxygen uptake during steep temperature decline. Under furnace-assisted cooling, uranium remained as UO_2_, which indicates that extended residence at higher temperature suppressed further oxidation. At 70 cm, within the thermal plateau, condensation entered a regime governed by the coupled influence of redox conditions and volatility. Both CeO_2_ and Ce_2_O_3_ were identified, which shows partial reduction of cerium during sustained high-temperature residence. Cesium appeared in limited amounts, consistent with its volatility. By 90 cm, the impact of thermal history was most apparent. Under ambient cooling, condensates were irregular and aggregated, and contained α-UO_3_, Cs_2_UO_4_, and minor Cs_2_O. These products reflect oxidation during continuous cooling and delayed, limited incorporation of cesium. In contrast, furnace-assisted cooling produced discrete and often layered particles containing Ce_2_O_3_, Cs_2_UO_4_, Cs_2_U_2_O_7_, and UO_2_. The persistence of Cs_2_U_2_O_7_ indicates that extended residence near 1400 K stabilized more cesium-rich uranates that would otherwise degrade or volatilize during ambient cooling. These differences show that high-temperature residence reshapes both redox evolution and the structure of condensed cesium-bearing products.

Bulk ICP-MS results corroborate these nanoscale trends. Near the reactor exit, Cs/Ce and U/Ce ratios converged to unity, which indicates complete condensation and stoichiometric closure. Upstream, large deviations were observed within and immediately downstream of the furnace plateau. Apparent Cs/Ce and Cs/U transient enrichments reflect a combination of delayed cesium condensation and preferential deposition of volatile Cs vapor relative to refractory U and Ce oxides. Deposition of U and Ce on upstream reactor walls further increases the apparent Cs enrichment at intermediate positions. Even when these effects are considered, the axial ICP-MS trends remain consistent with the nanoscale observations showing suppressed cesium condensation within the plateau and subsequent incorporation during downstream cooling.

Collectively, these TEM and ICP-MS measurements describe a coherent view of how thermal gradients modify condensation pathways. Uranium and cerium form near-equilibrium refractory oxides under both regimes. Cesium follows a kinetically constrained path governed by volatility and residence time. It remains in the vapor under high-temperature conditions, enriches transiently within the plateau, and stabilizes downstream as mixed Cs–U oxides. Equilibrium models predict the dominant refractory oxides but do not capture these apparent transient deviations or the formation of metastable and mixed U–Cs products. Whether these mixed oxides form or persist under different chemical compositions or elemental ratios in this simplified system remains an active area of investigation. Nevertheless, incorporating kinetic factors such as residence time, local thermal gradients, and volatility is essential for improving predictive models of multicomponent condensation. The combined cross-scale observations confirm that the plasma flow reactor provides a controlled setting to isolate how temperature gradients influence redox evolution, volatility-driven fractionation, and the stabilization or degradation of condensed phases. This framework helps determine where near-equilibrium assumptions hold and where kinetic corrections are required for accurate description of high-temperature condensation in complex chemical matrices.

### Relevance for Understanding Condensation in Fallout-like Systems

Although the PFR does not reproduce the full chemical complexity of a nuclear fireball, it isolates key processes that influence late-stage cooling and the redistribution of volatile and refractory species. The controlled thermal gradients provide a means to evaluate how residence time and temperature history shape redox evolution and chemical fractionation. Within this simplified U/Ce/Cs system, cesium shows the strongest sensitivity. Its condensation is delayed, its distribution evolves during extended high-temperature residence, and it enriches transiently before stabilizing as stable, mixed Cs–U oxides further downstream. Uranium and cerium condense within narrower temperature windows and remain closely associated throughout cooling, although uranium continues to oxidize under prolonged ambient cooling. These observations show that sustained high temperature followed by rapid quenching produces transient volatility-driven enrichments that later move toward stoichiometric closure. This capability provides a direct way to determine how thermal history influences when volatile elements interact with a refractory matrix, which is central to interpreting fractionation signatures in nuclear debris.

The formation of Cs-uranate phases in this system raises questions about their relevance to natural fireball conditions. It remains uncertain whether these same compounds would form in an actual nuclear cloud. Real fireballs cool rapidly and often entrain substantial environmental material, particularly in surface-burst tests, where fission products and actinides dissolve into a silicate melt that quenches far from equilibrium.^1,6,7,10^ The present experiments therefore provide a controlled platform for probing the delay in cesium condensation relative to refractory elements, without overextending these findings to the full complexity of real fallout. The appearance of reduced Ce­(III) phases under furnace-assisted conditions illustrates the sensitivity of multivalent systems to high-temperature residence. Ce­(III) oxides tend to be less volatile than Ce­(IV), which may promote their local condensation and retention within the condensed phase.^37–39^ This behavior differs from that of uranium, whose higher oxidation states generally remain more volatile under oxidizing conditions.^40,43^ Similar redox–volatility relationships have been reported for plutonium,^44–46^ yet in nuclear melt glass it is predominantly Pu­(IV) because Fe-bearing phases act as a redox buffer.^13^ These comparisons show that modest shifts in temperature or oxygen fugacity can influence the relative volatility and retention of multivalent actinides and rare-earth elements and help explain the divergent behavior of uranium and cerium under the thermal conditions imposed in the reactor.

Overall, these findings highlight that even modest shifts in redox potential or thermal gradient can influence the partitioning of volatile and refractory species. The PFR provides a controlled setting for examining how thermal gradients influence redox evolution and condensate structure. These mechanistic insights are not intended to replicate the full chemical or physical complexity of a nuclear detonation. Instead, they provide the foundational understanding needed to interpret more complex high-temperature experiments in multicomponent matrices.

## Conclusions

These experiments demonstrate that controlled thermal gradients in the plasma flow reactor modify both redox evolution and cesium partitioning in the ternary U/Ce/Cs system. Uranium and cerium condensed early as UO_2_ and CeO_2_, consistent with equilibrium-controlled oxide formation. Under ambient cooling, uranium oxidized to α-UO_3_, while furnace-assisted hold-up preserved UO_2_ and promoted partial reduction of cerium to Ce_2_O_3_. Cesium showed the strongest kinetic sensitivity. It remained volatile upstream, displayed transient enrichment within the thermal plateau, and stabilized downstream as mixed Cs–U oxides, including Cs_2_UO_4_ and Cs_2_U_2_O_7_. The formation of these Cs-uranate phases highlights how volatility, residence time, and redox conditions together influence late-stage condensation. Whether such phases would form or persist under lower cesium concentrations or in more chemically complex matrices remains uncertain and is the focus of ongoing work.

The broader insight is that phase stability and element partitioning depend not only on equilibrium thermodynamics but also on the local temperature and redox environment experienced during cooling. The agreement between nanoscale TEM results and bulk ICP-MS measurements provides a cross-scale view of how thermal history shapes condensate evolution. The findings confirm that equilibrium models capture the dominant refractory oxides but do not describe the processes that govern volatile-element redistribution. Incorporating parameters such as residence time, volatility, and local thermal gradients is therefore essential for improving predictive descriptions of multicomponent condensation.

Although the reactor cannot reproduce the full chemical complexity of a nuclear fireball, it provides a controlled platform for isolating mechanisms that delay or advance interaction between volatile and refractory components. This capability strengthens efforts to interpret fractionation signatures in simplified debris systems. The experimental framework developed here can be extended to more complex chemistries, including systems containing silicate or environmental components. Future efforts that integrate in situ diagnostics and kinetic modeling will help refine how volatility, redox chemistry, and temperature history together influence condensate structure and the evolving composition of multicomponent debris formed under high temperature, redox-evolving conditions.

## Supplementary Material



## Notes

Detailed experimental protocols for ICP-MS analysis, including alumina stub collection and digestion procedures, a schematic of the alumina collection hardware (Figure S1) and additional axial fractionation data for Cs/Ce, U/Ce, and Cs/U under ambient and furnace-assisted thermal regimes (Figures S2 and S3) (PDF)

## References

[1] Klement, J. Radioactivefallout from Nuclear Weapons Tests, Proceedings of the Second Conference, Germantown, Maryland, November 3–6, 1964. AEC Symposium Series No. 5; CONF-765; Division of Biology and Medicine (AEC): Washington, D.C., 1965.

[2] Adams C. E., Farlow N. H., Schell W. R. (1960). The Compositions,
Structures and
Origins of Radioactive Fall-out Particles. Geochim.
Cosmochim. Acta.

[3] Crocker G. R., O’Connor J. D., Freiling E. C. (1966). Physical and Radiochemical
Properties
of Fallout Particles. Health Phys..

[4] Bellucci J. J., Simonetti A., Koeman E. C., Wallace C., Burns P. C. (2014). A Detailed
Geochemical Investigation of Post-Nuclear Detonation Trinitite Glass
at High Spatial Resolution: Delineating Anthropogenic vs. Natural
Components. Chem. Geol..

[5] Freiling E. C. (1961). Radionuclide
Fractionation in Bomb Debris. Science.

[6] Tompkins, R. C. ; Russell, I. J. ; Nathans, M. W. A Comparison between Cloud Samples and Close-In Ground Fallout Samples from Nuclear Ground Bursts. In Radionuclides in the Environment.; American Chemical Society, 1970; Vol. 93, pp 381–400 10.1021/ba-1970-0093.ch021.

[7] Freiling E. C., Kay M. A. (1966). Radionuclide Fractionation in Air-Burst Debris. Nature.

[8] Dardenne, Y. M. X. M. ; Parker, W. E. ; Knight, K. B. Chemical Fractionation is Not a Constant: Revisiting Bomb Vapor Chemistry, USDOE, 2020.

[9] Moody, K. J. ; Grant, P. M. ; Hutcheon, I. D. Nuclear Forensic Analysis; CRC Press: Boca Raton, 2005 10.1201/9780203507803.

[10] Weisz D. G., Jacobsen B., Marks N. E., Knight K. B., Isselhardt B. H., Matzel J. E. (2018). Diffusive Mass Transport
in Agglomerated Glassy Fallout
from a Near-Surface Nuclear Test. Geochim. Cosmochim.
Acta.

[11] Lewis L. A., Knight K. B., Matzel J. E., Prussin S. G., Zimmer M. M., Kinman W. S., Ryerson F. J., Hutcheon I. D. (2015). Spatially-Resolved
Analyses of Aerodynamic Fallout from a Uranium-Fueled Nuclear Test. J. Environ. Radioact..

[12] Cassata W. S., Prussin S. G., Knight K. B., Hutcheon I. D., Isselhardt B. H., Renne P. R. (2014). When the Dust Settles: Stable Xenon
Isotope Constraints
on the Formation of Nuclear Fallout. J. Environ.
Radioact..

[13] Pacold J. I., Lukens W. W., Booth C. H., Shuh D. K., Knight K. B., Eppich G. R., Holliday K. S. (2016). Chemical Speciation of U, Fe, and
Pu in Melt Glass from Nuclear Weapons Testing. J. Appl. Phys..

[14] Koroglu B., Mehl M., Armstrong M. R., Crowhurst J. C., Weisz D. G., Zaug J. M., Dai Z., Radousky H. B., Chernov A., Ramon E., Stavrou E., Knight K., Fabris A. L., Cappelli M. A., Rose T. P. (2017). Plasma Flow Reactor
for Steady State Monitoring of Physical and Chemical Processes at
High Temperatures. Rev. Sci. Instrum..

[15] Koroglu B., Wagnon S., Dai Z., Crowhurst J. C., Armstrong M. R., Weisz D., Mehl M., Zaug J. M., Radousky H. B., Rose T. P. (2018). Gas Phase Chemical
Evolution of Uranium,
Aluminum, and Iron Oxides. Sci. Rep..

[16] Kautz E. J., Weerakkody E. N., Finko M. S., Curreli D., Koroglu B., Rose T. P., Weisz D. G., Crowhurst J. C., Radousky H. B., DeMagistris M., Sinha N., Levin D. A., Dreizin E. L., Phillips M. C., Glumac N. G., Harilal S. S. (2021). Optical
Spectroscopy and Modeling of Uranium Gas-Phase Oxidation: Progress
and Perspectives. Spectrochim. Acta, Part B.

[17] Koroglu B., Mehl M., Crowhurst J. C., Zaug J. M., Rose T. P., Radousky H. B., Armstrong M. R. (2019). Experimental
and Modeling Study of
Chemical-Based Strategies for Mitigating Dust Formation in Fusion
Reactors. Plasma Phys. Controlled Fusion.

[18] Koroglu B., Dai Z., Finko M., Armstrong M. R., Crowhurst J. C., Curreli D., Weisz D. G., Radousky H. B., Knight K. B., Rose T. P. (2020). Experimental Investigation of Uranium Volatility during
Vapor Condensation. Anal. Chem..

[19] Rodriguez K., Weerakkody E. N., Dai Z., Knight K. B., Koroglu B., Rose T. P., Balboni E. (2023). Influence of Temperature History
and Flow Mixing on the Vapor-Phase Speciation of Uranium Oxide Nanoparticles. ACS Earth Space Chem..

[20] Koroglu B., Finko M., Saggese C., Wagnon S., Foster S., McGuffin D., Lucas D., Rose T. P., Crowhurst J. C., Weisz D. G., Radousky H. B., Curreli D., Knight K. B. (2022). The Influence
of Cooling Rate on Condensation of Iron, Aluminum, and Uranium Oxide
Nanoparticles. J. Aerosol Sci..

[21] Weerakkody E. N., Koroglu B., Dai Z., Rodriguez K. E., Balboni E. (2024). Dependence of Uranium Oxide Polymorphism
on Plasma
Synthesis Conditions. J. Phys. Chem. A.

[22] Rodriguez K., Koroglu B., Hammons J., Dai Z., Ferrier M. G., Balboni E., Rose T., Knight K. B. (2022). Vapor-Phase Aggregation
of Cerium Oxide Nanoparticles in a Rapidly Cooling Plasma. ACS Earth Space Chem..

[23] Sweet L. E., Corbey J. F., Gendron F., Autschbach J., McNamara B. K., Ziegelgruber K. L., Arrigo L. M., Peper S. M., Schwantes J. M. (2017). Structure
and Bonding Investigation of Plutonium Peroxocarbonate
Complexes Using Cerium Surrogates and Electronic Structure Modeling. Inorg. Chem..

[24] Klamm B. E., Windorff C. J., Marsh M. L., Meeker D. S., Albrecht-Schmitt T. E. (2018). Schiff-Base
Coordination Complexes with Plutonium­(IV) and Cerium­(IV). Chem. Commun..

[25] Ochiai A., Imoto J., Suetake M., Komiya T., Furuki G., Ikehara R., Yamasaki S., Law G. T. W., Ohnuki T., Grambow B., Ewing R. C., Utsunomiya S. (2018). Uranium Dioxides
and Debris Fragments Released to the Environment with Cesium-Rich
Microparticles from the Fukushima Daiichi Nuclear Power Plant. Environ. Sci. Technol..

[26] Yasunari T. J., Stohl A., Hayano R. S., Burkhart J. F., Eckhardt S., Yasunari T. (2011). Cesium-137 Deposition
and Contamination of Japanese
Soils Due to the Fukushima Nuclear Accident. Proc. Natl. Acad. Sci. U.S.A..

[27] Takaku Y., Higaki S., Hirota M., Kagi H. (2023). Radiocesium-Bearing
Microparticles Found in Dry Deposition Fallout Samples Immediately
after the Fukushima Nuclear Accident in the Kanto Region, Japan. Sci. Rep..

[28] Desgranges L., Baldinozzi G., Rousseau G., Nièpce J.-C., Calvarin G. (2009). Neutron Diffraction Study of the in Situ Oxidation
of UO2. Inorg. Chem..

[29] Zachariasen W. H. (1948). Crystal
Chemical Studies of the 5f-Series of Elements. I. New Structure Types. Acta Crystallogr..

[30] Ackermann R. J., Chang A. T., Sorrell C. A. (1977). Thermal
Expansion and Phase Transformations
of the U3O8–z Phase in Air. J. Inorg.
Nucl. Chem..

[31] Vickery R. C. (1947). Variation
in Density of Cerium Oxide. Nature.

[32] Bevan D. J.
M. (1955). Ordered
Intermediate Phases in the System CeO2—Ce2O3. J. Inorg. Nucl. Chem..

[33] Tsai K.-R., Harris P. M., Lassettre E. N. (1956). The Crystal
Structure of Cesium Monoxide. J. Phys. Chem.
A.

[34] Van
den Berghe S., Verwerft M., Laval J.-P., Gaudreau B., Allen P. G., Van Wyngarden A. (2002). The Local Uranium Environment in
Cesium Uranates: A Combined XPS, XAS, XRD, and Neutron Diffraction
Analysis. J. Solid State Chem..

[35] Bedford, R. G. ; Jackson, D. D. Volatilities of the Fission Product and Uranium Oxides, UCRL-12314; Lawrence Radiation Lab., Univ. of California: Livermore (US), 1965.

[36] Langowski, M. H. ; Darab, J. G. ; Smith, P. A. Volatility Literature of Chlorine, Iodine, Cesium, Strontium, Technetium, and Rhenium; Technetium and Rhenium Volatility Testing, PNNL--11052, PVTD--C95-02-03G, 211388; 1996; p PNNL--11052, PVTD--C95-02-03G, 211388; IAEA, 1996.

[37] Han Z.-K., Liu W., Gao Y. (2025). Advancing the Understanding of Oxygen Vacancies in
Ceria: Insights into Their Formation, Behavior, and Catalytic Roles. JACS Au.

[38] Xu Y., Zhou Y., Li Y., Liu Y., Ding Z. (2024). Advances in
Cerium Dioxide Nanomaterials: Synthesis Strategies, Property Modulation,
and Multifunctional Applications. J. Environ.
Chem. Eng..

[39] Gangopadhyay S., Frolov D. D., Masunov A. E., Seal S. (2014). Structure
and Properties
of Cerium Oxides in Bulk and Nanoparticulate Forms. J. Alloys Compd..

[40] Adachi G.-y., Imanaka N. (1998). The Binary Rare Earth Oxides. Chem. Rev..

[41] Cordfunke E. H. P., Van Egmond A. B., Van Voorst G. (1975). Investigations
on Cesium Uranates—I: Characterization of the Phases in the
Cs—U—O System. J. Inorg. Nucl.
Chem..

[42] Mijlhoff F. C., Ijdo D. J. W., Cordfunke E. H. P. (1993). The
Crystal Structure of α-
and β-Cs2U2O7. J. Solid State Chem..

[43] Deblonde G. J.-P., Kersting A. B., Zavarin M. (2020). Open Questions on the Environmental
Chemistry of Radionuclides. Commun. Chem..

[44] Romanchuk A. Y., Vlasova I. E., Kalmykov S. N. (2020). Speciation
of Uranium and Plutonium
From Nuclear Legacy Sites to the Environment: A Mini Review. Front. Chem..

[45] Ikeda-Ohno A., Shahin L. M., Howard D. L., Collins R. N., Payne T. E., Johansen M. P. (2016). Fate of Plutonium
at a Former Nuclear Testing Site
in Australia. Environ. Sci. Technol..

[46] Kersting A. B., Efurd D. W., Finnegan D. L., Rokop D. J., Smith D. K., Thompson J. L. (1999). Migration of Plutonium in Ground Water at the Nevada
Test Site. Nature.

