# Supplementary material for: Thermal Gradient Effects on Redox Evolution and Volatility-Driven Fractionation in Ternary U/Ce/Cs Condensates
Source: Anal Chem. 2026 Apr 24;98(18):13469–78. doi: 10.1021/acs.analchem.5c07929 (PMC13177278; doi:10.1021/acs.analchem.5c07929)
Supplement: Supplementary file 1 [file ac5c07929_si_001.pdf]

Supporting Information

Thermal Gradient Effects on Redox Evolution and Volatility-Driven Fractionation in Ternary U/Ce/Cs Condensates

Rakia Dhaoui,* Emily N. Weerakkody, Timothy P. Rose, Batikan Koroglu, Enrica Balboni

Physical and Life Sciences, Lawrence Livermore National Laboratory, Livermore, California 94550, United States

*Corresponding Author: Dr. Rakia Dhaoui, Email: dhaoui1@llnl.gov.

Table of Contents

1. Experimental Procedure for ICP-MS Analysis ……………………………………………..……… S3

1.1. Figure S1 ………………..……………………………………..…...…………..………… S3

2. Alumina Stub Digestion Protocol ……………………….……………….………………… S3–S4

3. Additional Elemental Fractionation Trends from ICP-MS Analysis ………….…………… S4

3.1. Figure S2 ………..……………………………………………………………….……… S4

3.2. Figure S3 ...…………………………………………………………………….………… S5

1. Experimental Procedure for the ICP-MS Analysis of U/Ce/Cs Condensates

A particle collection method was developed to obtain samples for inductively coupled plasma–mass spectrometry (ICP-MS) analysis. Because ICP-MS requires analytes to be prepared in 2% HNO_3, the procedure was designed to dissolve collected particles without dissolving the substrate. Al_2O_3 stubs were chosen as substrates for their thermal stability. The collection assembly consisted of an alumina tube, an alumina adapter, and the alumina stub (Figure S1). The assembly was inserted into the plasma flow reactor (PFR) tube and positioned at the desired distance from the radiofrequency (RF) coil. Before each run, the stub was preheated in the reactor for 5 min with the plasma operating and no analyte injection. A U/Ce/Cs nitrate solution was then introduced for 5 min, after which the plasma was extinguished and the stub cooled for 5 min. Stubs were removed, placed in clean 2 mL centrifuge tubes, and stored for subsequent etching and dissolution. Prior to experiments with U/Ce/Cs mixtures, an $Fe(NO_3)_3$ solution was used to validate the method. SEM-EDS confirmed the presence of iron oxide particles on the stub surface, demonstrating that alumina substrates were effective for particle collection under the extreme reactor conditions.

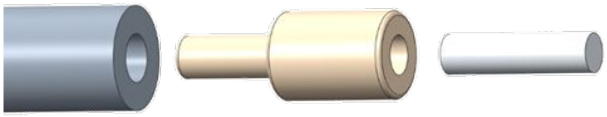

Figure S1. Computer-aided design (CAD) illustration of alumina stub collection hardware. From left to right: alumina tube (~1.25 m) for insertion into the PFR, alumina adapter piece, and alumina stub substrate for particle collection. The plasma region is located to the right of the image.

2. Protocol for Alumina Stub Digestion for ICP-MS Analysis

Each alumina stub was transferred to a clean Savillex vial. Residual particulates from the sampling assembly were recovered by rinsing the original centrifuge tubes with 0.5 mL of 9 M hydrochloric acid (HCl), followed by agitation and transfer to the corresponding Savillex vial. An additional 2.0 mL of 9 M HCl was added to each vial, which was then capped and heated at 80°C for approximately 22 hours to ensure complete etching of the sample surface and dissolution of metal oxides. After digestion, the solution was transferred to a new Savillex vial. The stub was rinsed with 0.5 mL of 9 M HCl, and the rinse was combined with the digested solution. The total volume (~2.5 mL) was evaporated to dryness on a hot plate at ~100 °C under a fume hood. To remove any residual matrix or organic contaminants, two drops (~0.2 mL) of concentrated nitric

acid (HNO_3) were added to each sample and again evaporated to dryness. This HNO_3 addition and drying step was repeated two additional times. Finally, each sample was reconstituted in 2 mL of 2% HNO_3, capped, and reheated overnight at ~80 °C to ensure complete solubilization. Once cooled, the solutions were transferred to 15 mL acid-cleaned centrifuge tubes for ICP-MS analysis. To verify the fidelity of the digestion and analysis protocol, separate aqueous standards containing uranyl, cerium, and cesium nitrates were prepared in the same 1:1:1 atomic ratio as the experimental inputs. These standards were digested using the same method and analyzed in parallel to confirm the input ratios and validate analytical recovery.

3. Additional Elemental Fractionation Trends from ICP-MS Analysis

Figure S2 provides a complementary view of fractionation by comparing Cs/U and Ce/U ratios as a function of temperature. These results reinforce that uranium and cerium condense together, while cesium diverges strongly under furnace-assisted conditions. Under ambient cooling (**Figure S2a**), Ce/U ratios remained steady (~0.6–1.1), consistent with co-condensation and minimal fractionation. Cs/U ratios showed broader variability (~0.8–1.6), indicating partial decoupling of cesium from the U/Ce condensation front but no large enrichment. In contrast, furnace-assisted conditions (**Figure S2b**) produced significant divergence. Cs/U ratios increased sharply along the reactor axis, reaching values up to 15. This pronounced enrichment highlights the sensitivity of cesium to extended high-temperature residence, which delays its condensation and favors kinetic trapping or stabilization in downstream phases.

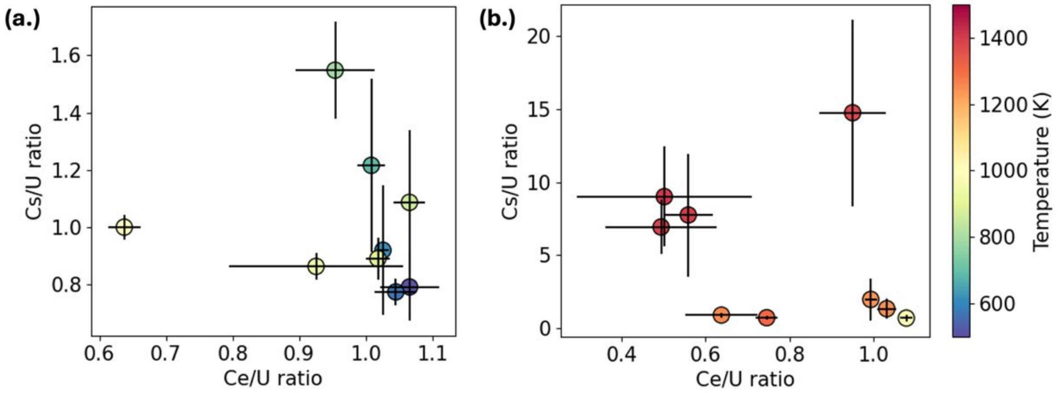

Figure S2. Colormap of Cs/U vs. Ce/U atomic ratios for the **(a)** ambient cooling condition and **(b)** furnace-assisted condition. Results reinforce Cs enrichment under thermal hold-up and consistent Ce/U behavior across regimes. Error bars represent one standard deviation from three independent replicate experiments (n = 3).

Figure S3 provides an additional view of these axial trends. Under ambient conditions (**Figure S3a**), Cs/U ratios converged toward unity below ~800 K, consistent with co-condensation and the absence of major perturbations. Under furnace-assisted conditions (**Figure S3b**), however, Cs/U ratios showed a sharp rise and subsequent drop when entering and exiting the thermal plateau, underscoring how sensitive cesium behavior is to residence time and thermal gradients.

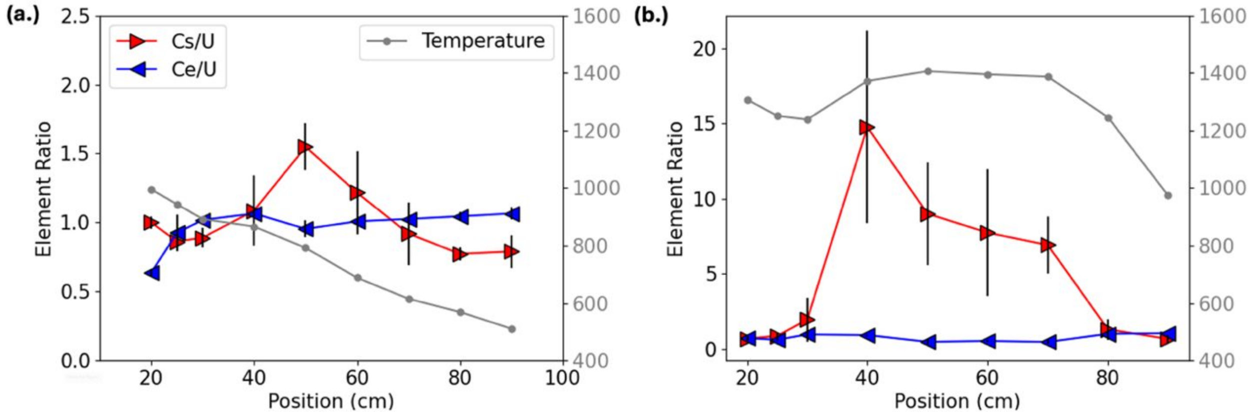

Figure S3. Axial profiles of Cs/U (*red forward arrows*) and Ce/U (*blue reverse arrows*) elemental ratios with temperature overlay (*gray points*) for **(a)** ambient and **(b)** furnace-assisted conditions. Error bars represent one standard deviation from three independent replicate experiments (n = 3).
